# Local administration of irinotecan using an implantable drug delivery device stops high-grade glioma tumor recurrence in a glioblastoma tumor model

**DOI:** 10.1007/s13346-024-01524-x

**Published:** 2024-02-06

**Authors:** Dina Abdelnabi, Sarah Lastakchi, Colin Watts, Hannah Atkins, Shawn Hingtgen, Alain Valdivia, Christopher McConville

**Affiliations:** 1 School of Pharmacy, Robert Aitken Institute for Clinical Research, Institute of Clinical Sciences, College of Medical and Dental Sciences, University of Birmingham, Edgbaston, B15 2TT UK; 2 Department of Neurosurgery, University Hospitals Birmingham, NHS Foundation Trust, Birmingham, UK; 3 Department of Pathology and Laboratory Medicine, School of Medicine, The University of North Carolina at Chapel Hill, Chapel Hill, NC 27599 USA; 4 Division of Molecular Pharmaceutics, UNC Eshelman School of Pharmacy, The University of North Carolina at Chapel Hill, Chapel Hill, NC 27599 USA

**Keywords:** Glioblastoma, Implantable device, Irinotecan, Local drug delivery

## Abstract

The treatment for Glioblastoma is limited due to the presence of the blood brain barrier, which restricts the entry of chemotherapeutic drugs into the brain. Local delivery into the tumor resection margin has the potential to improve efficacy of chemotherapy. We developed a safe and clinically translatable irinotecan implant for local delivery to increase its efficacy while minimizing systemic side effects. Irinotecan-loaded implants were manufactured using hot melt extrusion, gamma sterilized at 25 kGy, and characterized for their irinotecan content, release, and drug diffusion. Their therapeutic efficacy was evaluated in a patient-derived xenograft mouse resection model of glioblastoma. Their safety and translatability were evaluated using histological analysis of brain tissue and serum chemistry analysis. Implants containing 30% and 40% w/w irinotecan were manufactured without plasticizer. The 30% and 40% implants showed moderate local toxicity up to 2- and 6-day post-implantation. Histopathology of the implantation site showed signs of necrosis at days 45 and 14 for the 30% and 40% implants. Hematological analysis and clinical chemistry showed no signs of serious systemic toxicity for either implant. The 30% implants had an 80% survival at day 148, with no sign of tumor recurrence. Gamma sterilization and 12-month storage had no impact on the integrity of the 30% implants. This study demonstrates that the 30% implants are a promising novel treatment for glioblastoma that could be quickly translated into the clinic.

**Graphical Abstract:**

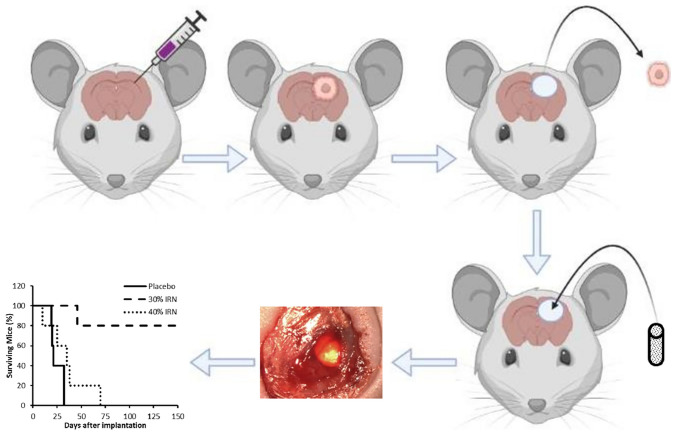

## Introduction 

The World Health Organization (WHO) classifies glioblastoma as a grade IV cancer due to its highly aggressive nature [1, 2]. It is the most common type of high-grade glioma and is characterized as genetically heterogenous, with an age-standardized global incidence rate of 4.6 per 100,000 population, per year [3]. The median overall survival for glioblastoma patients is just 14.6 months [4, 5], with no improvement despite advances in neuroimaging, surgery, radiotherapy, and chemotherapy. The introduction of 5-aminolevulinic (5ALA)-based fluorescence-guided neurosurgery has improved rates of gross total resection and increased progression-free survival; however, infiltrative tumor tissue remains within the adjacent brain parenchyma and is responsible for tumor recurrence [6, 7].

The standard treatment for glioblastoma is surgical resection, followed by radiotherapy and subsequent treatment with 150–200 mg/m^2^ of temozolomide [8]. Temozolomide’s ability to penetrate the blood brain barrier (BBB) is a limiting factor in the efficacy of this treatment [9, 10]. The BBB is a limiting factor in the efficacy of systemic treatment for glioblastoma, restricting therapeutic concentrations being achieved within the glioblastoma margin, with dose-limiting systemic toxicities resulting in the development of glioblastoma resistance [11, 12].

Local drug delivery into the resection cavity at the time of surgery would allow for the administration of high local doses to the margin, with reduced systemic toxicities. One such approach is the Gliadel wafer, containing 3.85% carmustine (BCNU), which demonstrated a modest effect on overall survival in randomized phase III trials [13, 14]. However, a Cochrane report demonstrated no significant clinical benefit for newly diagnosed glioblastoma patients, with a small but significant clinical benefit to recurrent glioblastoma patients [15]. Their awkward shape and placement onto the margin mean that BCNU suffers from poor drug penetration into the tumor margin and does not reach the deep-seated tumor tissue.

We previously demonstrated that IRN-loaded implants containing 30% or 40% w/w of irinotecan (IRN) prolonged survival in a glioblastoma xenograft mouse resection model with no sign of tumor recurrence and mild to moderate local toxicity [16]. IRN is a semi-synthetic pro-drug [17], whose active metabolite 7-ethyl-10-hydroxycamptothecin (SN-38) acts as an inhibitor of the topoisomerase I group of enzymes [18]. Topoisomerase I enzymes act within the cell to induce temporary breaks within one or both strands of DNA, allowing the DNA to uncoil for transcription and replication [19]. Topoisomerase I forms a covalent linkage with DNA, allowing it to form a cleavable complex [19]. SN-38 binds to topoisomerase I in this confirmation, inhibiting the enzymes from re-joining the strands of DNA and causing S-phase-specific cell killing [18–20]. IRN is used in the treatment of advanced colorectal cancer in combination with 5-fluorouracil (5-FU) and folinic acid [21].

IRN has had a mixed response as a monotherapy for gliomas with some studies demonstrating efficacy [22–24], while others have demonstrated no response [25–28]. There was no consistency of dose across all studies, while Gilbert et al. demonstrated that the concurrent administration of enzyme-inducing antiepileptic drugs (EIAEDs) changed the pharmacokinetics of IRN including its toxicities and efficacy [29]. In combination with temozolomide (TMZ), IRN was shown to be active and tolerable [30, 31], while in combination with carmustine, IRN was shown to be active and non-cross-resistant in recurrent glioblastoma patients after treatment with TMZ [32]. A Phase I dose-escalating study recommended doses of IRN for a phase 2 clinical trial when given in combination with carmustine (100 mg/m^2^) of 225 mg/m^2^ for patients on EIAEDs and 125 mg/m^2^ for patients not on EIAEDs [33]; however, the subsequent Phase II study demonstrated no benefit when compared to IRN alone [34]. In combination with bevacizumab, IRN was generally determined to be an active regimen with acceptable toxicity [35–43]. In general, a review by Vredenburgh et al. demonstrated that IRN monotherapy has demonstrated efficacy; however, its efficacy appears to be enhanced when used in combination with other chemotherapeutic agents [20]. When administered concurrently with EIAEDs, the dosage must be increased to compensate for enhanced cytochrome CY3A4/5 enzyme activity [20]. Toxicities associated with irinotecan have been manageable; the most important dose-limiting toxicities are neutropenia and diarrhea, concluding that IRN-based chemotherapy of malignant glioma merits further study [20]. IRN crosses the BBB at high intravenous doses between 125 and 500 mg/m^2^; however, these doses result in serious systemic side effects particularly gastrointestinal toxicity, leading to early and late-onset diarrhea, and severe neutropenia, which has limited IRN’s use as a treatment for gliomas [44].

Our research group clinically evaluated the local delivery of IRN to the brain for the treatment of recurrent glioblastoma using Drug Eluting Beads (DEBs) in a Phase I clinical study (identifier: NCT02433392) [45]. The results from this study were promising with an absence of swelling, and inflammation normally seen with Gliadel®. None of the patients demonstrated the normal systemic drug-related toxicities associated with IRN [45]. This study demonstrated that local delivery of IRN into the brain parenchyma offered a safer route of administration over systemic delivery in the treatment of glioblastoma. However, the DEBs were only capable of delivering IRN for up to 72 h, while most of the DEB gel formulation was pushed out of the brain parenchyma and into the bed of the resection cavity. Therefore, the use of solid implant that could deliver IRN directly into the tumor margin over a sustained period compared to the DEBs could improve the clinical performance of IRN as a treatment for glioblastoma.

Due to both IRN and poly (lactic-co-glycolic acid) (PLGA) having been administered directly into the human brain, an IRN/PLGA implant could be translated very quickly into the clinic. The aim of this study is to build on our previous IRN Drug Eluting Seed (iDES) work [16] to aid the translation of the IRN/PLGA implant into the clinic. This study initially investigates the removal of the plasticizer, due to it never having been administered directly into the brain, the in vitro release and diffusion of 30% and 40% w/w IRN-loaded PLGA implants. Our previous study used a U87 mouse resection model to assess the efficacy of the iDES [16]. However, given the lack of invasiveness in this model, it is not considered clinically relevant. Therefore, in this study, the 30% and 40% implants were subsequently investigated for their in vivo toxicity and efficacy using a highly invasive, clinically relevant patient-derived xenograft (PDX) glioblastoma mouse resection model [46, 47]. Wakimoto et al. demonstrated that this model produced tumors with no distinctive border that extended through the corpus callosum to the contralateral brain, proving its highly aggressive and invasive nature making it an excellent representation of human glioblastoma [46]. Finally, the impact of gamma sterilization, accelerated and long-term storage conditions on the IRN content, release, distribution, and crystallinity in the 30% implants was investigated.

## Materials and methods

### Materials

The poly (lactic-co-glycolic acid) (PLGA) DLG 4A 50:50 lactide to glycolide ratio polymer (Lakeshore biomaterials) was purchased from Corbion (Amsterdam, Netherlands). Irinotecan hydrochloride was purchased from LGM Pharma (Nashville, TN). Acetonitrile, dichloromethane, DMSO, methanol, and sodium phosphate were purchased from Sigma-Aldrich (Dorset, England).

### Manufacture of 30% and 40% w/w IRN-loaded implants

The PLGA and IRN were weighed, roll mixed for 10 min, fed into a 7.5 mm 16:1 twin-screw extruder (Kapex Manufacturing LLC, Saginaw, MI, US) at a feed rate of 140 g per hour and a screw speed of 60 RPM, extruded through a 2 mm die, and cut into 6 × 2 mm implants weighing approximately 24 mg.

### Determination of the IRN content of the 30% and 40% w/w IRN-loaded implants

A sample (*n* = 10) of the 30% or 40% w/w IRN-loaded implants was weighed into a glass vial containing dichloromethane (3 mL) and left for 1 h to dissolve. The vials were placed in a water bath at 60 °C to evaporate the dichloromethane, 3 mL of mobile phase was added to precipitate out the PLGA, and the vials were placed into an ultrasonic bath for 1 min, then placed into an orbital shaking incubator (Unitron HT infors) at 37 °C and 60 rpm overnight, filtered, and analyzed by HPLC.

### Irinotecan HPLC methodology

HPLC analysis was isocratic and performed on an Agilent 1200 HPLC with a Phenomenex Luna C18 4.6 × 150 mm column with a 5-µm particle size. The mobile phase comprised of 75% phosphate buffer (pH of 2.7) and 25% acetonitrile. The flow rate was 1.00 mL/min, and UV detection was performed at a wavelength of 225 nm with an injection volume of 20 µL. Linearity was found to be in the range of 0.01 to 10 mg/mL with an *R*^2^ of 1.00.

### In vitro accelerated release of IRN from the 30% and 40% w/w IRN-loaded implants

The IRN-loaded implants (*n* = 6) were placed into 3 mL of DMSO to MeOH (10:90) in an orbital shaking incubator at 37 °C and 60 rpm. Complete media replacement was performed at days 1, 2, 3, 5, and 7, and the samples were mixed with 3 mL of mobile phase, filtered, and analyzed via HPLC.

### In vitro release of IRN from the 30% and 40% w/w IRN-loaded implants

The 30% and 40% w/w IRN-loaded implants (*n* = 6) were placed into sealed vials containing 3 mL of deionized water in an orbital shaking incubator at 37 °C and 60 rpm. Complete media replacement was performed at days 1, 2, 5, 7, 9, 13, 17, 21, 26, 30, 34, and 40. The samples were mixed with 3 mL of mobile phase, filtered, and analyzed via HPLC.

### In vitro diffusion of IRN from the 30% and 40% w/w IRN-loaded implants

0.6% agarose gels were prepared in a Petri dish (8 cm in diameter) and the 30% and 40% w/w IRN-loaded implants were implanted in the center. The Petri dishes were wrapped with Parafilm to prevent water evaporation and placed in an agitated incubator at 37 °C. Samples of gel were removed at 5, 10, 20, and 30 mm from the seed on days 1, 3, 5, and 7, weighed, dissolved in 2 mL of DMSO, mixed with 1 mL of mobile phase, filtered, and analyzed via HPLC. Brain tissue and 0.6% agarose gel have a similar density of 1.075 g/cm^3^. This density value was used to convert the weight of the gel into mL and subsequently used to calculate the concentration of IRN in the gel.

### Gamma sterilization of the placebo, 30%, and 40% w/w IRN-loaded implants

The placebo, 30%, and 40% w/w IRN-loaded implants were individually placed into sealed aluminum pouches and sterilized using Caesium-137 radiation at 25 kGy on dry ice to assure a low temperature during the irradiation process.

### In vivo evaluation of the toxicity of the 30% and 40% w/w implants in sham resection cavities of non-tumor bearing mice

To assess their toxicity, the 30% and 40% w/w IRN-loaded implants were implanted into sham resection cavities of non-tumor bearing immunocompetent C57/BL6 mice (Charles River Laboratories). A mixture of males and females aged 8–10 weeks was used for the toxicity study. The toxicity study was approved by the University of North Carolina at Chapel Hill Institutional Animal Care and Use Committee (IACUC). The mice were monitored and the degree of pain, using the mouse grimace scale, as well as rough hair, weight loss, dehydration, edema, swelling, and itching were individually scored on a scale of 0–4.99%, no toxicity; 5–33.3%, mild toxicity; 33.4–66.6%, moderate toxicity; 66.7–100%, severe toxicity and summed to achieve a percent “toxicity score” for each implant at each time point. One-hundred-fifty-microliter blood samples were taken at days 4, 14, and 45 and hematological assessed for hemoglobin, reticulocytes, lymphocytes, neutrophils, basophils, white blood cells, and eosinophils levels. Clinical chemistry was performed on the blood to determine blood urea nitrogen, creatinine, alkaline phosphatase, alanine transaminase, and aspartate aminotransferase levels as an assessment of kidney and liver function. Mice were sacrificed via transcardial perfusion at days 4, 14, and 45 post-implantation. Brains were stored in 10% formalin upon collection and then moved to 2.5% formalin after 24 h. Brains were cut in coronal orientation at the rostral and caudal edges of the resection cavity and then embedded in paraffin blocks. Blocks were sectioned into 4-µm thick slices and stained with H&E. The resulting histological slides were examined by a blinded clinical pathologist and the percent toxicity scored on a scale of 0–20%, no toxicity; 21–30%, mild toxicity; 31–50%, moderate toxicity; and 51–100%, severe toxicity. The in vivo toxicity study adhered to the NIH Guide for the Care and Use of Laboratory Animals.

### In vivo efficacy testing of the 30% and 40% w/w IRN-loaded implants in a patient-derived xenograft glioblastoma mouse resection model

To assess their efficacy, the 30% and 40% w/w IRN-loaded implants were implanted into resection cavities of tumor-bearing immunocompetent C57/BL6 mice (Charles River Laboratories). A mixture of males and females aged 8–10 weeks was used for the efficacy study. The efficacy study was approved by the University of North Carolina at Chapel Hill IACUC. All biological samples implanted into animals were approved by the University of North Carolina at Chapel Hill Institutional Biosafety Committee (IBC). Assuming that 50% of the control mice will have recurrence within 2 weeks but that fewer than 10% of the treated mice will, using 5 mice in each of the groups will provide at least 80% power using a two-sided test at the 0.05 significance level. We anticipate a 20% attrition rate due to surgery, bringing the total to six mice per group. Orthotopic glioblastoma tumors were established in the brains of immunodeficient athymic nude mice (*n* = 6 in each group) via stereotaxic injection. Briefly, 450 × 10^6^ cells of patient-derived glioblastoma cells expressing mCherry-Fluc in 5 µl serum-free DMEM were loaded into a 10 µl capacity Hamilton syringe. The needle was positioned at stereotaxic coordinates (AP 0.0, ML 2.5, DV − 2.0) from the bregma point. Tumor cells were then injected at 1 µl/min and allowed to settle for 5 min, and then, the needle was retracted at 0.5 mm/min. The tumors were given 1 week to engraft and grow, and the mice were randomly assigned to treatment groups. Established tumors were then resected under fluorescent guidance, and the implants were implanted into the resulting resection cavity. Changes in tumor volume were tracked by bioluminescence. Mice were injected with 150 mg/kg luciferin IP and then imaged 10 min later in an IVIS Kinetic imager under isoflurane anesthesia. Identically sized regions of interest were drawn over the heads of each mouse, and average radiance was recorded. For tumor measurements, experimenters were not blinded (as it is commonly accepted in the field). The mice were monitored for survival and Kaplan–Meier survival curves were produced. Upon death/sacrifice, the whole brains of the mice were removed, stored in 10% formalin upon collection, and then moved to 2.5% formalin after 24 h. The brains were imaged using bioluminescence.

### Stability of the 30% w/w IRN-loaded implants under accelerated and long-term storage conditions

Sterilized and unsterilized 30% w/w IRN-loaded implants in aluminum pouches were placed in a stability chamber (Binder KBF constant climate chamber) at 25 °C and 40% relative humidity, as well as a refrigerator at 4 °C with samples (*n* = 6) taken at 0, 1, 3, 6, and 12 months. The samples were assessed for their IRN content, accelerated release, distribution, and crystallinity.

### Raman spectroscopy mapping of a cross-section of the sterilized and unsterilized 30% w/w IRN-loaded implants

Raman spectra, in the Raman shift range of 3200–200 cm^−1^, were collected using a dispersive DXR Raman Microscope (Thermo Fisher Scientific). Spectra were collected using a laser, with an excitation wavelength of 780 nm, and its respective optical filter and diffraction grating. The 780-nm laser had a 20-mW maximum power at the sample. The microscope with a 10 × objective was used and a motorized stage was used for the mapping experiments. The obtained spectra were pre-processed by Automatic Baseline Correction before running Multivariate Curve Resolution (MCR) coupled with RGB (Red, Green, Blue) display in the Omnic Software package. MCR analyzed the data and attempted to produce spectra that represented the user-defined number of pure components. MCR and RGB also provided spatial distributions of each component. IRN was designated as red, while PLGA was designated as green.

### Powder X-ray diffraction (PXRD) analysis of the sterilized and unsterilized 30% w/w IRN-loaded implants after 0, 1, 3, 6, and 12 months on accelerated storage

The implants were milled into a powder form and PXRD analysis was performed using a Panalytical Empyrean diffractometer (PANanalyical, Almelo, The Netherlands) with Cu Kα radiation (*λ* = 1.54060) at 40 kV and 40 mA between 5 and 80° (2 θ) at 25 °C.

### Differential Scanning Calorimeter (DSC) analysis of the sterilized and unsterilized 30% w/w IRN-loaded implants after 0, 1, 3, 6, and 12 months on accelerated storage

DSC analysis was performed to determine the level of crystallinity of IRN in the sterilized and unsterilized 30% w/w IRN-loaded implants after 0, 1, 3, 6, and 12 months on real-time and accelerated storage. The implants were milled into a powder form and the DSC thermographs were recorded using a Discovery DSC-25 (TA Instruments, Newcastle, UK). Ten-milligram samples were heated in aluminum pans over the temperature range of 25–300 °C at a constant rate of 10 °C/min under a nitrogen purge (50 mL/min). The enthalpy value for IRN in the 30% w/w IRN-loaded implants was divided by the enthalpy value for 3 mg of pure IRN (10.72 J/g) and multiplied by 100 to calculate its % crystallinity within the implants.

### Statistical analysis

Sample size was predetermined from pilot experiments and/or experiments that have been done in the past, to obtain statistically significant data. Experiments were repeated at least once. Replicates were reproducible. Statistical analysis was performed using a one-way analysis of variance (ANOVA) (GraphPad Prism version 5.02 for Windows, GraphPad Software, San Diego, CA). Post-hoc comparisons of the means were performed using Tukey’s Honestly Significance Difference test. A significance level of *P* < 0.05 was accepted to denote significance in all cases. The significance between groups in the Kaplan–Meier survival analysis was determined by the chi-square test.

## Results

### Characterization of the 30% and 40% w/w IRN-loaded implants without plasticizer

**Fig. 1:**
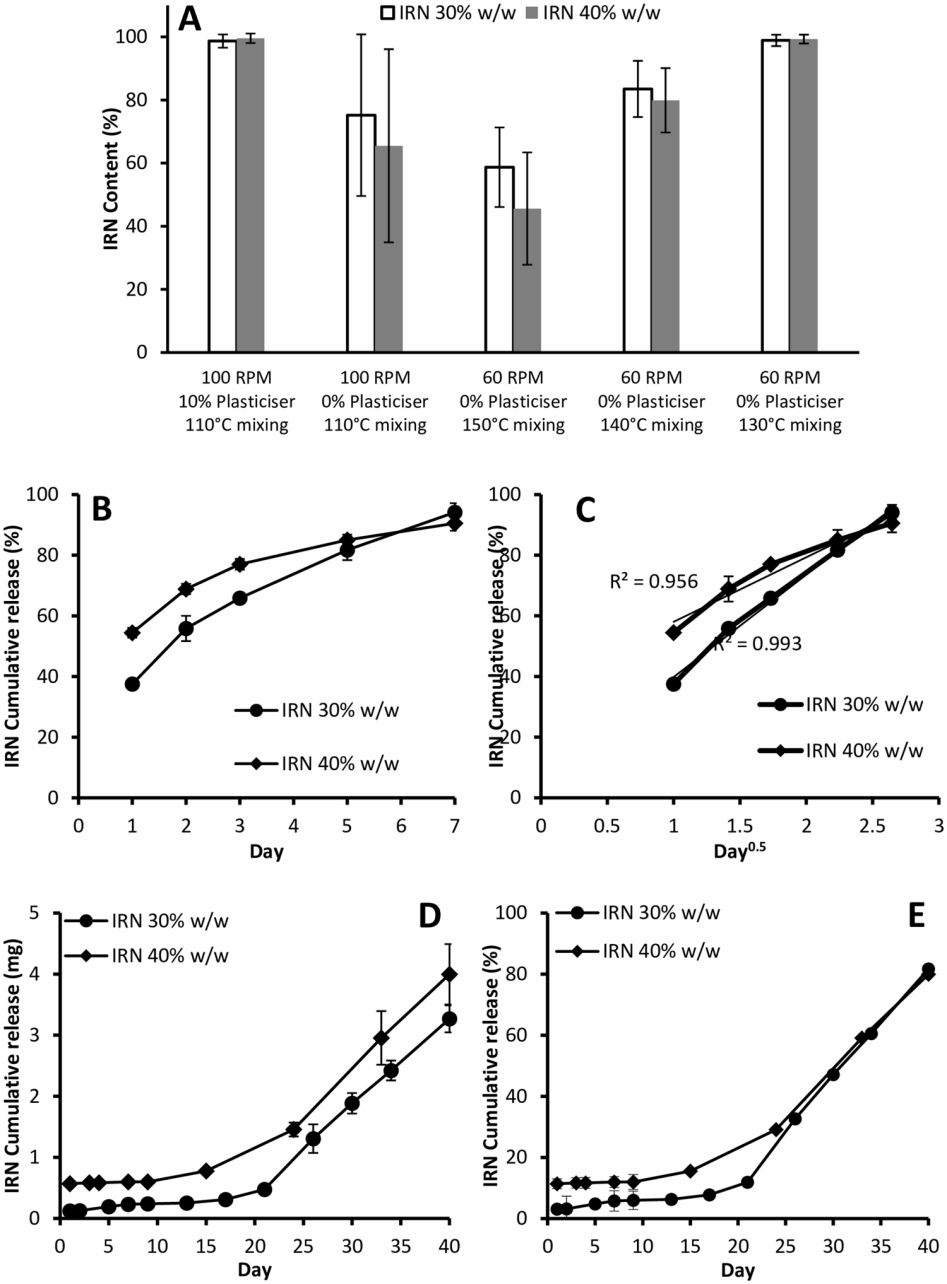
IRN content with and without the use of plasticizer and under various HME conditions (**A**). In vitro accelerated dissolution cumulative release vs time profiles for the 30% and 40% w/w IRN-loaded implants (**B**). In vitro accelerated dissolution release versus the square root of time profiles for the 30% and 40% w/w IRN-loaded implants (**C**). In vitro cumulative release (**D**) and percent release (**E**) versus time for the 30% and 40% w/w IRN-loaded implants. *n* = 6 for all figures and each figure shows the mean ± the standard deviation

The previously published IRN-loaded implants contained 10% w/w of the Kolliphor P 188 plasticizer and were extruded using a mixing zone temperature of 110 °C and a screw speed of 100 RPM [16]. To aid translation into the clinic, we were advised to remove the plasticizer as it has never been administered directly into the brain. Figure 1A demonstrates that the removal of the plasticizer resulted in a significant (*P* = 0.02) reduction and variation in the IRN content. Reducing the screw speed to 60 RPM and increasing the mixing temperature to 150 °C further reduced the IRN content; however, the variation in content was decreased. Decreasing the temperature to 140 °C increased the IRN content and reduced the variation, while decreasing the temperature further to 130 °C resulted in an acceptable IRN content and variation.

Both the 30% and 40% implants released 70% of their IRN content within 7 days (Fig. 1B), while their cumulative release versus the square root of time profiles confirms that they both obey matrix release kinetics having a linear cumulative release versus square root of time profile (Fig. 1C). The in vitro cumulative release (Fig. 1D) and percent release (Fig. 1E) demonstrate that both the 30% and 40% w/w IRN-loaded implants have the potential to release IRN for at least 40 days, with both implants releasing 80% of their IRN content by day 40 (Fig. 1E).

#### IRN diffusion from the implants

**Fig. 2:**
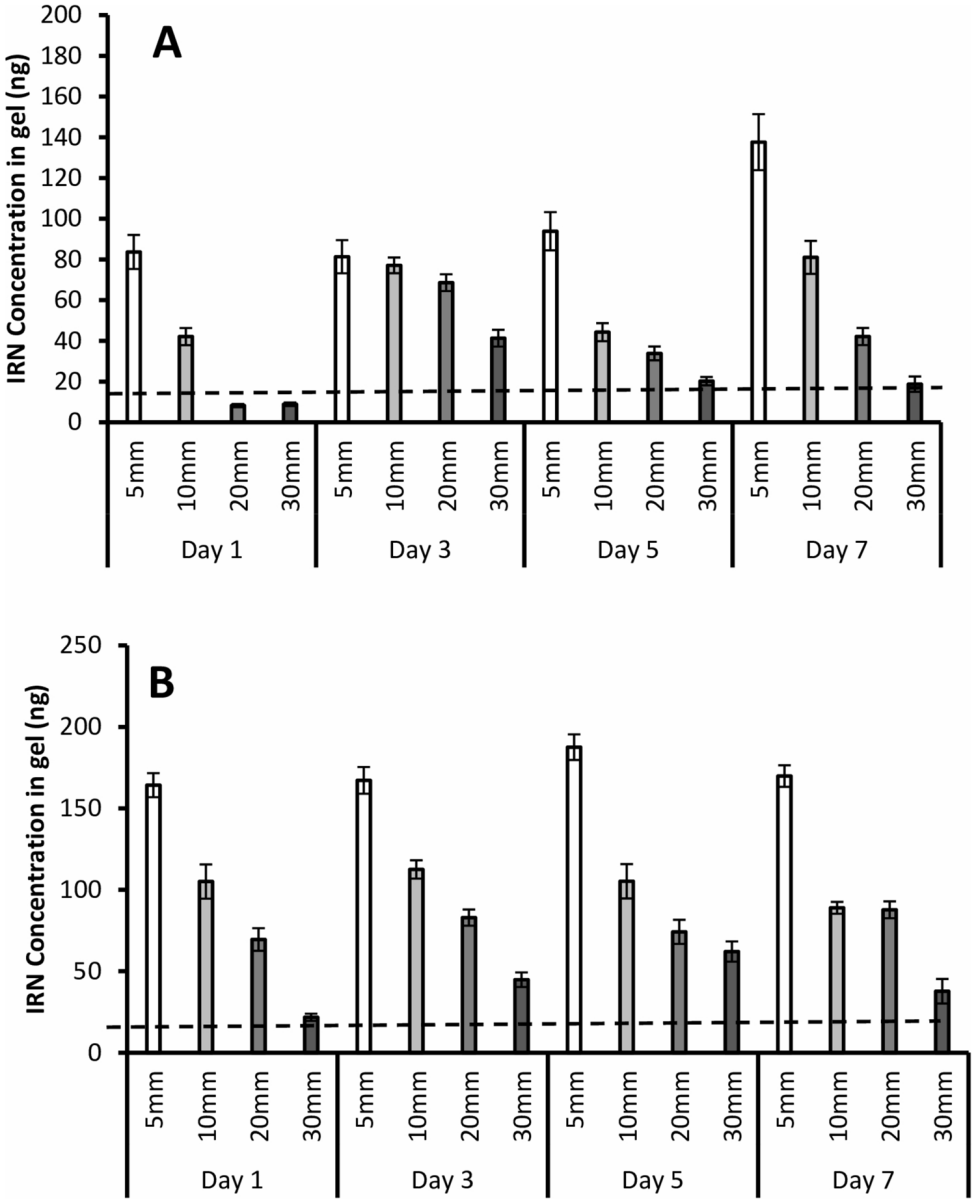
Diffusion of IRN from the 30% w/w (**A**) and 40% w/w (**B**) IRN-loaded implants. The dashed line indicates the IC_50_ of IRN against primary glioblastoma cells [16]. *n* = 6 for all figures and each figure shows the mean ± the standard deviation

Agarose gels at concentrations of 0.4 to 0.6% have similar mechanical properties and density (1.075 g/cm^3^) to those of brain tissues [48]. Figure 2 demonstrates that greater amounts of IRN were detected at all distances across all days for the 40% implants compared to the 30% implants (*P* values 0.01 to 0.03). Both implants demonstrated the same trend with decreasing IRN levels further from the implantation site and increasing levels from day 1 to 7 (Fig. 2). The 30% implants had IRN levels below the IC_50_ value at 20 and 30 mm from the implantation site on day 1, increasing to above the IC_50_ value by day 3 (Fig. 2A), while the 40% implants had IRN levels above the IC_50_ value at all distances and across all days (Fig. 2B).

#### In vivo toxicity of the 30% and 40% w/w IRN-loaded implants

**Fig. 3:**
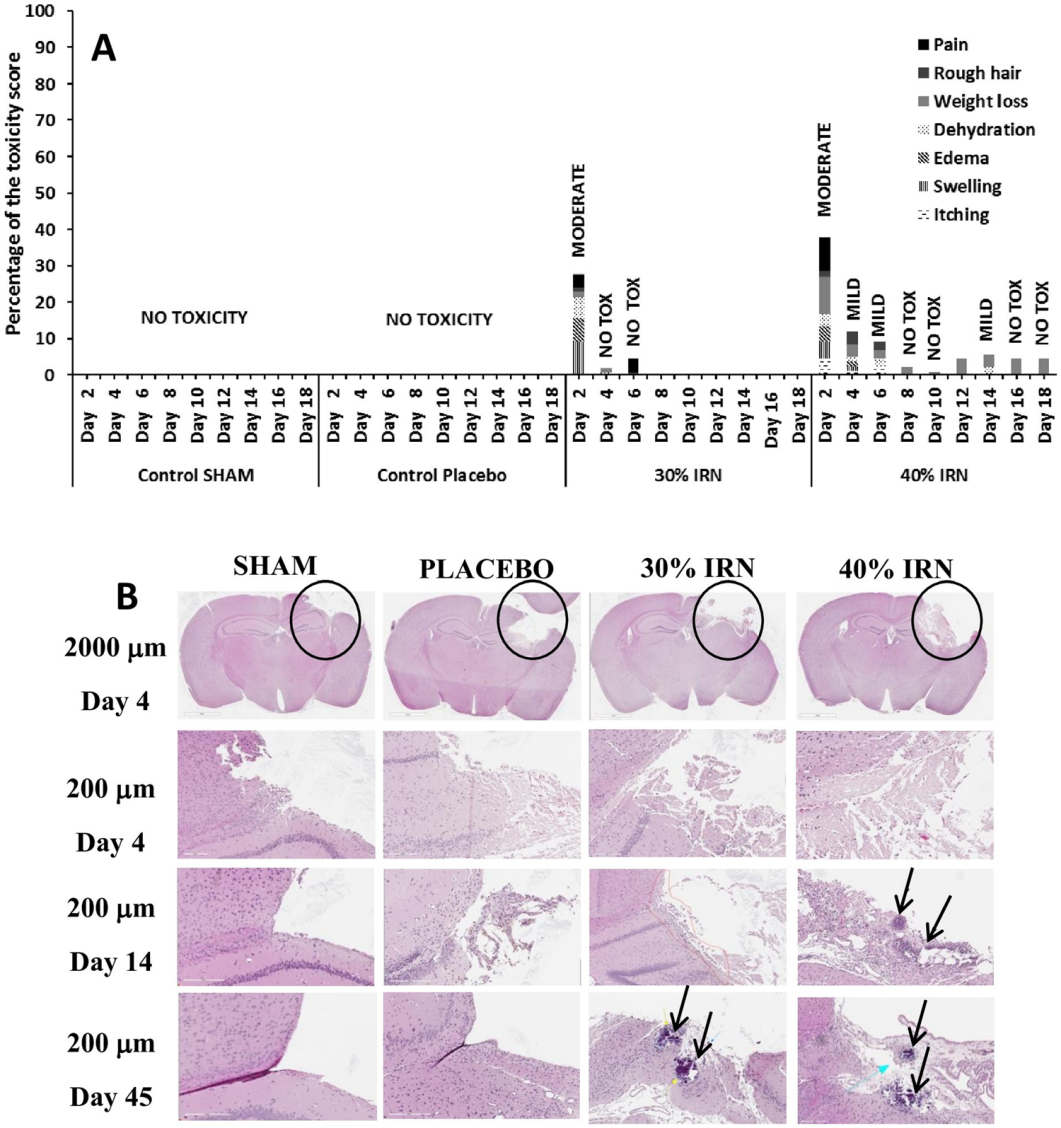
In vivo toxicity score of mice after sham surgery and the implantation of placebo, 30%, or 40% w/w IRN-loaded implants (**A**). H&E stained slides of the brain region around the resection/implantation site (circle) at days 4 and 14 and after sham surgery and the implantation of placebo, 30%, or 40% w/w IRN-loaded implants (**B**). The arrows indicate necrotic areas of the brain tissue

**Fig. 4:**
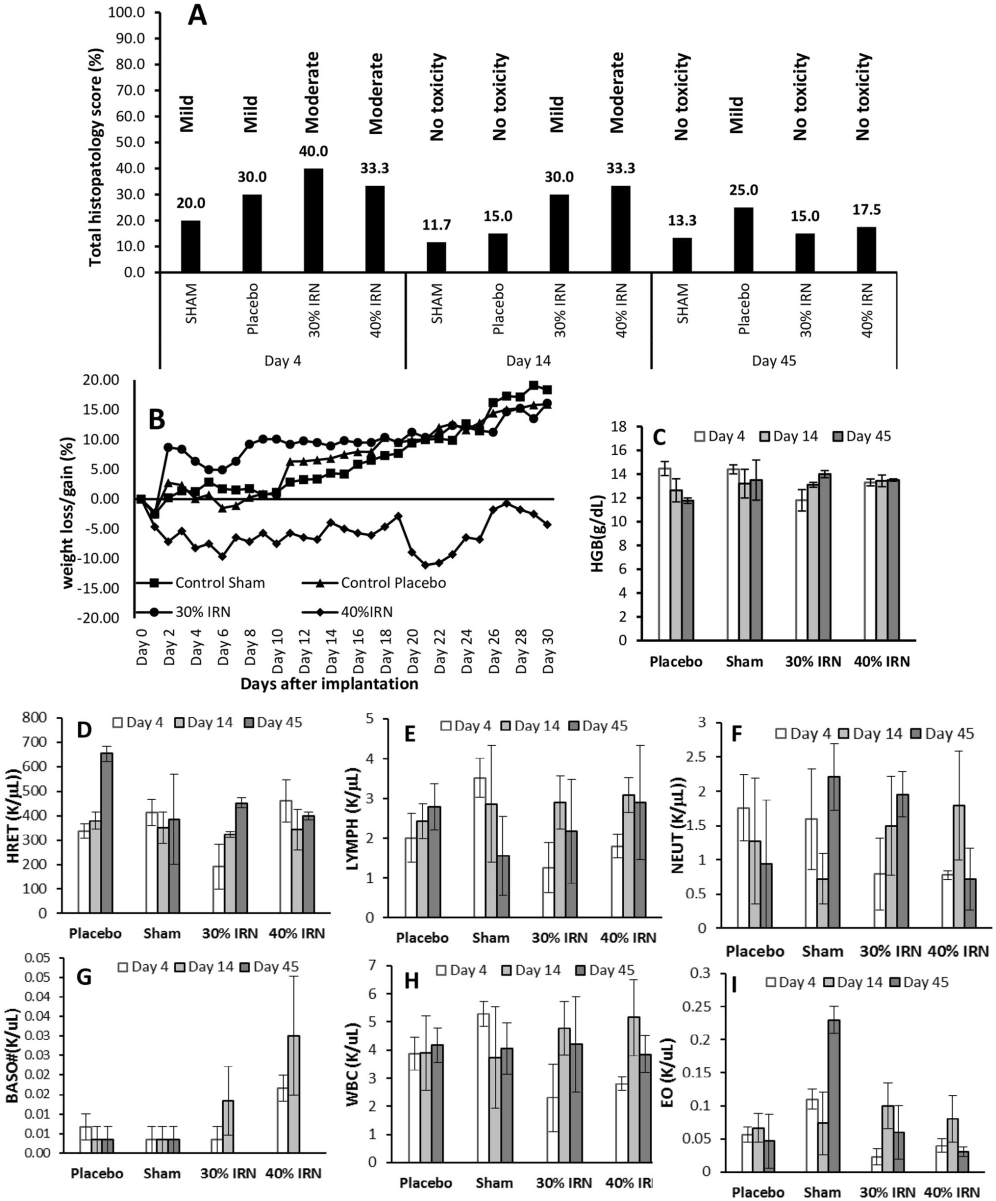
Histopathology score of the H&E stained brain tissue (**A**), percentage weight loss (**B**), and hematology assessment (hemoglobin, reticulocytes, lymphocytes, neutrophils, basophils, white blood cells, and eosinophils) of the mice (**C–I**) after sham surgery and the implantation of placebo, 30%, or 40% w/w IRN-loaded implants. *n* = 5 for all figures and **C** to **I** show the mean ± the standard deviation

The 30% implants demonstrated moderate toxicity on day 2, which disappeared by day 4 (Fig. 3A). The 40% implants showed signs of mild to moderate toxicity, including swelling, pain, and edema up to 6 days after implantation, which disappeared by day 8 with mild toxicity such as itching and swelling returning at day 14 and disappearing at day 16 (Fig. 3A). The H&E stained slides of the brain region around the resection/implantation site (Fig. 3B) show signs of necrosis at day 45 for the 30% implants and days 14 and 45 for the 40% implants. The early onset of necrosis for the 40% implants is due to their greater release of IRN during the first days after implantation (Figs. 1 and 2). The histopathology score (Fig. 4A) demonstrates mild to moderate local toxicity of the brain tissue at day 4 across all groups. The toxicity associated with the sham and placebo groups dissipates by day 14, with mild toxicity returning at day 45 for the placebo group. The 30% and 40% implant groups had mild and moderate toxicity at day 14, respectively, which disappeared by day 45. The 40% group had significant weight loss (Fig. 4B) when compared to the other groups, losing 9.6% of their body weight by day 6, when they started to gain weight; however, by day 21, they had lost 11.1%, when they began to gain weight again. The weight loss correlates with the toxicity scores (Fig. 3A), with the mice having mild to moderate toxicity up to 6 days after implantation, which diminished by day 8 with mild toxicity returning at day 14 and disappearing at day 16.

#### Hematological analysis

Figure 4C to I present the hemoglobin (HGB), reticulocytes, lymphocytes, neutrophils, basophils, white blood cells (WBCs), and eosinophils of the mice. Figure 4C demonstrates that for all of the groups, the HGB levels remained within the normal range of 12 and 17 g/dL. The reticulocyte levels (Fig. 4D) were high for all groups; however, elevated reticulocytes are associated with surgery. All groups had lymphocyte levels within the normal range of 1000 to 4800 lymphocytes per microliter (Fig. 4E). The neutrophil levels (Fig. 4F) of both the Sham and Placebo groups dropped from below the normal range (1500 and 8000 neutrophils per microliter) on day 14, increasing to normal levels by day 45, in the placebo group, while the sham group reduced further. With the 30% implant group, the neutrophil levels were below normal on day 4, increasing to the normal range by day 14. In the 40% group, the neutrophil levels were below the normal range on day 4, increasing to normal levels by day 14 and then dramatically decreasing to below the normal range by day 45. For the sham, placebo, and 30% groups, the basophil levels (Fig. 4G) remain within the normal range of 0 to 300 per microliter of blood over the 45 days. However, for the 40% group, the basophil levels were at the high end of the normal range on day 4 and above the normal range at day 14, returning to the normal range by day 45. Both the Sham and Placebo groups had WBC levels within the normal range (Fig. 4H) of 4000 to 11,000 per microliter of blood, while the WBC level for the 30% and 40% groups fell below the normal range on day 4, returning to the normal range by day 14. All groups had eosinophil levels within the normal range of 500 cells per microliter of blood (Fig. 4I). There was a significant increase in eosinophil levels for the sham surgery group at day 45; however, they remained within the normal range.

#### Clinical chemistry

**Fig. 5:**
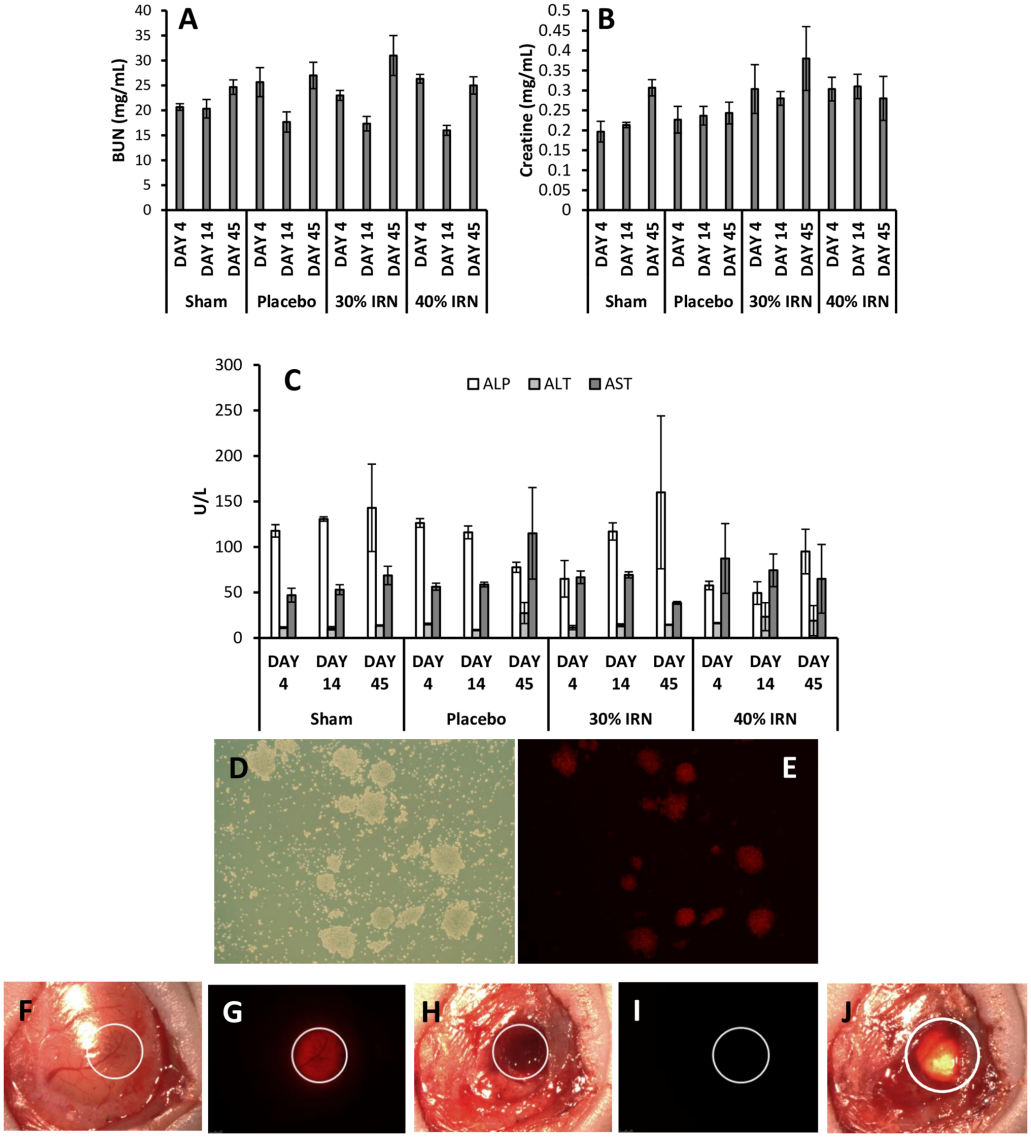
The measurement of Blood Urea Nitrogen (**A**), creatinine levels (**B**), alkaline phosphatase, alanine transaminase, and aspartate aminotransferase (**C**) levels in the blood. Primary glioblastoma cells labelled with mCherry-Fluc reporters for fluorescence microscopy and Bioluminescence imaging (**D** and **E**). Visualization of the intracranial window with the glioblastoma tumor cells implanted (**F**), the tumor cells visualized with the mCherry filter (**G**), the resection cavity (**H**), the resection cavity visualized under the mCherry filter to confirm no residual tumor (**I**), and the implant in the cavity (**J**). *n* = 5 for **A** to **C** and show the mean ± the standard deviation

Figure 5A and B assess kidney function through the measurement of blood urea nitrogen (BUN) and creatinine levels in the blood, respectively. Both the BUN and creatinine levels were in the normal range (6 to 30 mg/mL and 0.7 to 1.2 mg/dL of blood, respectively) for all treatment groups. Figure 5C assesses liver function by measuring alkaline phosphatase (ALP), alanine transaminase (ALT), and aspartate aminotransferase (AST) levels in the blood. The ALP and ALT levels of all groups were within the normal range of 44 to 147 and 4 to 36 U/L of blood, respectively. However, the AST levels in all groups were above the normal range (8 to 33 U/L of blood), which would suggest that it is not associated with IRN levels in the blood.

#### In vivo efficacy of the 30% and 40% w/w IRN-loaded implants using a patient-derived xenograft model of glioblastoma

Figure 5D and E show the primary glioblastoma cells labelled with mCherry-Fluc reporters for fluorescence microscopy and bioluminescence imaging, respectively. The tumors grow in spheres which means that upon xenotransplantation into the immunodeficient mice, they will form glioblastoma tumors which reflect the histopathological heterogeneity of the parental tumor [49, 50]. Figure 5F to J provide a visualization of the intracranial window with the glioblastoma tumor cells implanted, the tumor cells visualized with the mCherry filter, the resection cavity, the resection visualized under the mCherry filter to confirm no residual tumor, and finally the implant in the cavity.

**Fig. 6:**
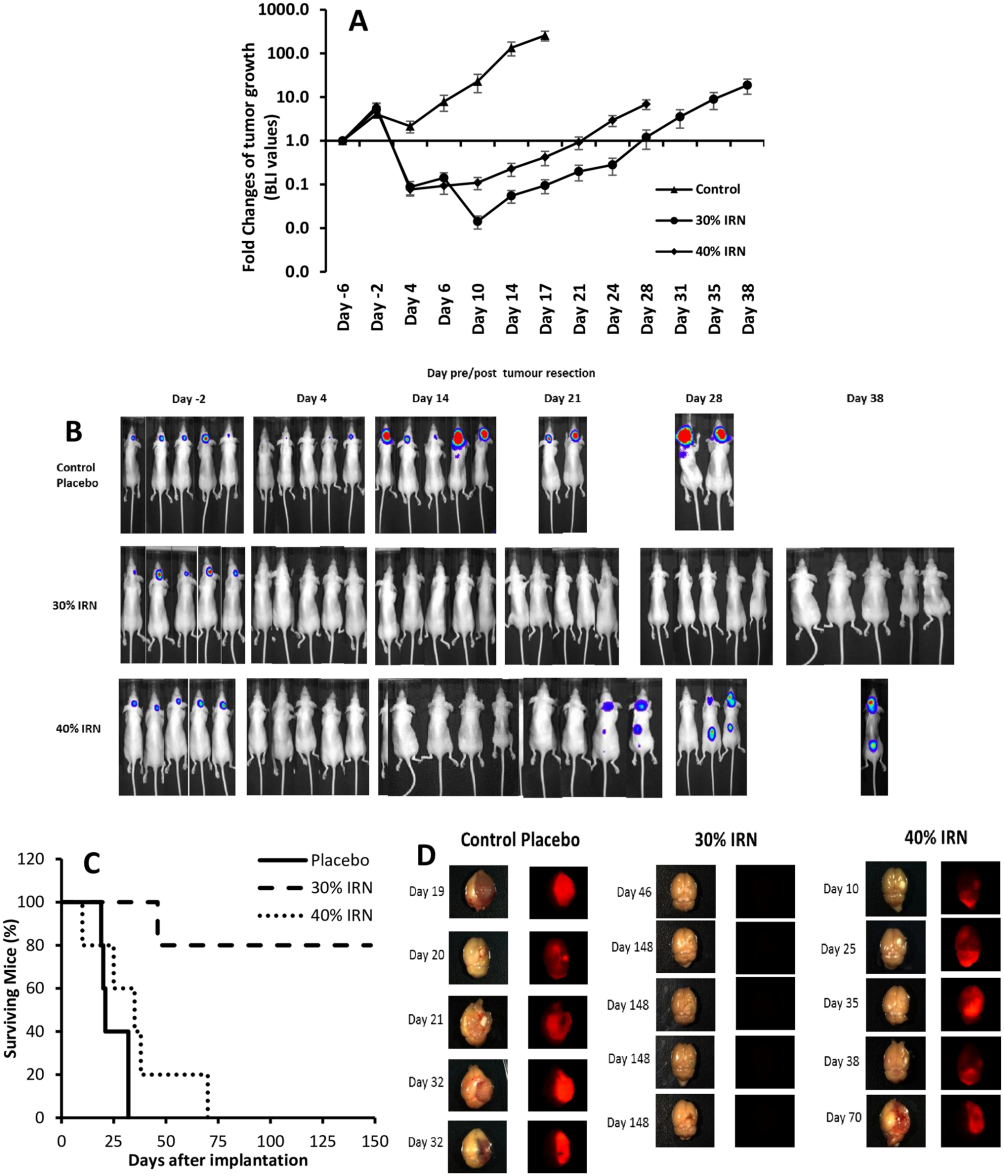
The fold change of bioluminescence imaging values for the placebo, 30%, and 40% w/w IRN-loaded implant groups (**A**). Fluc bioluminescence images of the mice at days 2 days pre-,  and 4, 14, 21, 28, and 38 days post-tumor resection (**B**). Kaplan–Meier survival curves (**C**) and bioluminescence images of the whole brains of the mice on the day they died or were euthanized (**D**) for the placebo, 30%, and 40% w/w IRN-loaded implant groups. *n* = 5 for **A** and shows the mean ± the standard deviation

The fold change of bioluminescence imaging (BLI) values for the placebo, 30%, and 40% w/w IRN-loaded implant groups is presented in Fig. 6A. All three groups had steady tumor growth from the day of tumor implantation (day − 6) to the day of tumor resection and implantation (day 0). After resection/implantation both the 30% and 40% groups demonstrated a reduction in tumor volume to below the baseline, whereas the placebo group had a significantly higher tumor volume that never went below the baseline and continued to increase until day 17. At day 4, the 40% w/w group demonstrated a slow tumor regrowth, reaching the baseline (1.0-fold) by day 21, while the 30% group had a further decrease in tumor volume until day 10 followed by a slow increase in tumor volume, reaching the baseline at day 28. Fluc BLI of the mice (Fig. 6B) demonstrated that all groups had tumors 2 days before resection surgery. The placebo group showed signs of tumor recurrence by day 4 post resection, with the BLI signal increasing by day 14 and 3 mice dying by day 18 with all mice dead by day 38. The 30% group demonstrated no signs of tumor recurrence by day 38, with all mice still alive. With the 40% group, there was tumor recurrence at day 21 in one of the mice, with signs of metastasis in another mouse. By day 28, two mice had died, with tumor recurrence and metastasis in the two of the surviving mice. At day 38, only one mouse was still alive, and it had both recurrent tumor and metastasis.

Figure 6C presents the survival data for each of the groups. In both the placebo and 40% groups, 100% of the mice died by day 32 and 70, respectively. However, with the 30% group, 80% of the mice were still alive at day 148, when they were euthanized. The brains of the dead mice were removed and imaged (Fig. 6D). The BLI images of the whole brains of the mice in the placebo and 40% group demonstrate that on each day, the mice died and they had tumor recurrence (Fig. 6D). However, with the 30% group, none of the mice had any sign of tumor recurrence at the time of death, even the mouse that died at day 46, and we believe that this death is not related to tumor recurrence.

#### Characterization of the impact of sterilization and storage on the stability of the 30% w/w IRN-loaded implants

**Fig. 7:**
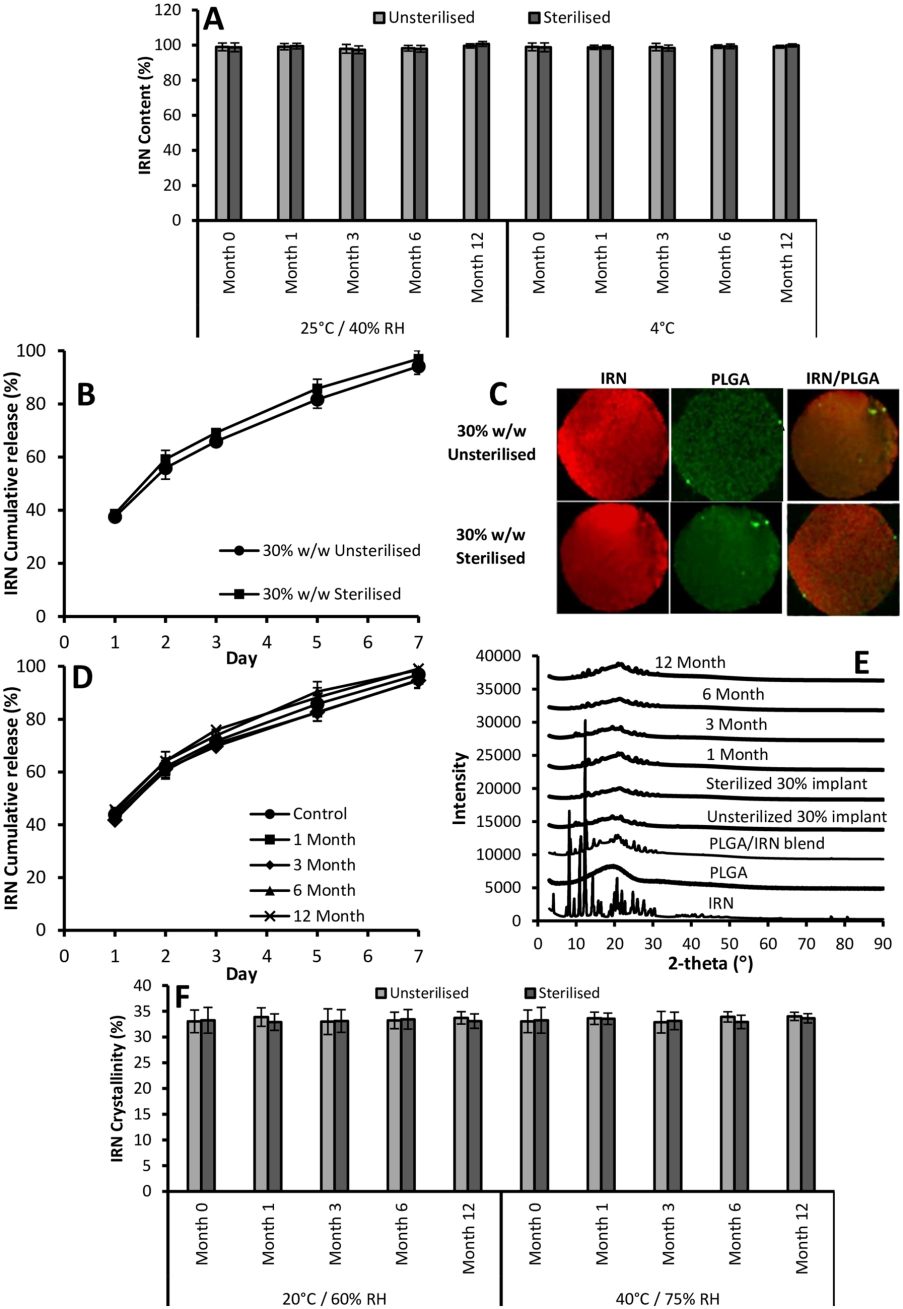
IRN content of the sterilized and unsterilized 30% implants after 0, 1, 3, 6, and 12 months under long-term (20 °C/60% RH) and accelerated (40 °C/75%) storage conditions (**A**). In vitro accelerated release profiles (**B**) and Raman maps of the cross-section (**C**) for the sterilized and unsterilized 30% implants. In vitro accelerated release profiles (**D**) and XRD patterns (**E**) for the sterilized 30% implants after 0, 1, 3, 6, and 12 months under long-term and accelerated storage conditions. IRN crystallinity of the sterilized and unsterilized 30% implants after 0, 1, 3, 6, and 12 months under long-term and accelerated storage conditions (**F**). *n* = 6 for **A**, **B**, **D,** and **F** with each figure showing the mean ± the standard deviation

Based on the toxicity and efficacy data, we believe that the 30% w/w IRN-loaded implants should be considered for translation into the clinic. One potential issue for the translation of PLGA-based implants is the impact of sterilization on their drug release and shelf-life. Gamma sterilization can impact the biodegradable polyesters, via the formation of reactive radicals, which may compromise the drug substance incorporated in the device and thus drug stability and release after irradiation need to be carefully evaluated [51–54]. Therefore, the 30% implants were sterilized and the impact of sterilization on their IRN content, release, distribution, crystallinity, and shelf-life under accelerated storage conditions for 12 months was determined. Figure 7A demonstrates that sterilization had no impact on the stability of the IRN within the implants (month 0) and that the IRN content remained stable under accelerated storage conditions for 12 months. Sterilization resulted in no significant (*P* = 0.15) impact on the release of IRN from the implants (Fig. 7B). Raman mapping of the cross-section of the implants (Fig. 7C) demonstrates that the sterilization process had no impact on the distribution of the IRN within the seeds. Furthermore, storing the 30% implants under accelerated storage conditions had no impact on their release of IRN (Fig. 7D) or the crystallinity of the IRN within the implants (Fig. 7D). The implants sampled at months 1, 3, 6, and 12 had similar release profiles to month 0, while the XRD analysis (Fig. 7E) shows no change in the level of IRN crystallinity within the implants at months 1, 3, 6, and 12, which is further supported by the percent crystallinity after DSC analysis (Fig. 7F).

## Discussion

We previously published IRN-loaded implants containing 10% w/w of the plasticizer Kolliphor P 188 [16]. However, to aid translation into the clinic, we were advised to remove the plasticizer as it has never been administered directly into the brain. To remove the plasticizer, we had to adjust the manufacturing process to produce implants with the correct drug content, diameter, and smooth surface. This was achieved by increasing the mixing temperature to 130 °C while reducing the screw speed (Fig. 1A). The higher mixing temperature reduces the viscosity of the PLGA, while the slower screw speed increases the residence time of the material in the extruder. This combination of lower viscosity and increased residence time improves the mixing and distribution of IRN within the PLGA. The lower viscosity reduces the die pressure, which removes the possibility of “shark skinning” resulting in a smooth surface. The slower screw speed and reduced die pressure result in the extrudate leaving the die at a slower rate, which ensures the diameter of the extrudate remains consistent to the size of the die orifice.

The accelerated dissolution study (Fig. 1B) demonstrates that both the 30% and 40% implants release 70% of their IRN content within 7 days. The 40% implants had a significantly (*P* = 0.01) greater release rate of IRN over the first 3 days of release, due to increased drug on their surface. Modelling the release data using the Higuchi equation (Fig. 1C) demonstrates that the release is diffusion-controlled, which is not surprising as they are matrix devices with IRN homogenously dispersed within the PLGA. The dissolution study (Fig. 1D) demonstrates that the 40% implants released significantly (*P* = 0.02) more IRN over 40 days compared to the 30% implants, due to more IRN on the surface of the implants leading to an increased release over the early stages of release. Adjusting for drug loading by comparing percentage release (Fig. 1E), the 40% implants still had a significantly greater release over the first 20 days of release, due to the increased release early on creating more pores in the PLGA allowing for the release media to imbibe into the seeds increasing both IRN release and PLGA degradation.

Brain tissue and 0.6% agarose gel have similar mechanical properties and densities (1.075 g/cm^3^) [48]. The IC_50_ of IRN against tumor margin tissue was shown to be 14.02 ng [16]. For both 30% and 40% implants, the tissue concentration of IRN decreased with an increase in the distance from the implantation site across all days (Fig. 2). However, the general trend was an increase in IRN concentration at all distances with an increase in time. For the 30% implants, all tissue concentrations were well in excess of the IC_50_ value [16] at 5 mm and 10 mm, while with the 40% implants, all tissue concentrations were well in excess of the IC_50_ up to 30 mm away from the implantation site. The significant increase in tissue concentrations for the 40% implants is due to it having a significantly greater release rate of IRN.

The 30% implants resulted in moderate toxicity on day 2 which disappeared by day 4 and necrosis on day 45. The 40% implants resulted in mild to moderate toxicity out to day 16 (Fig. 3A) and necrosis at day 14, which was still present at day 45 (Fig. 3B). The increased toxicity and necrosis associated with the 40% implants are due to their increased IRN release, particularly over the first 3 to 7 days (Fig. 1B and D), which results in higher IRN tissue levels (Fig. 2) increasing toxicity. The histopathology score (Fig. 4A) demonstrates mild to moderate local toxicity of the brain tissue at day 4 across all groups, which is associated with the surgery. The 30% and 40% implant groups had mild and moderate toxicity at day 14, respectively, associated with the release of IRN and which dissipated for both groups by day 45. The increased toxicity of the 40% implants had an impact on the weight of the mice, with them losing up to 11.1% of their weight over the first 3 weeks post-implantation. Despite the toxicity of the 40% implants being only moderate, it does highlight the importance of the IRN loading in the implants. Furthermore, the toxicity was observed over the first 2 to 3 weeks post-implantation. This is not surprising as the implants are matrix drug delivery devices and their increased early release rate will drive the IRN into the tissue, increasing tissue concentrations, which is important for efficacy, but can also result in local toxicity.

Systemic administration of IRN is associated with a high incidence of grade 1 or 2 anemia (49–60%) [55]; grade 3 or 4 anemia occurs in 8–10% of the patients [56]. Normal HGB levels are between 12 and 17 g/dL, while the WHO toxicity criteria for anemia consider grade 1 (mild) anemia to occur at HGB levels of 10.0 g/dL to within normal limits, with grade 2 (moderate) occurring between 8.0 to 10.0 g/dL, and while grade 3 (severe) and grade 4 (life-threatening) occur between 6.5 and 7.9 g/dL and less than 6.5 g/dL. The hematological analysis (Fig. 4C-I) demonstrates that for all of groups, the HGB levels remained within normal conditions. This would indicate that the IRN released from the implants did not enter the systemic circulation at sufficient concentration to result in anemia. The reticulocyte level for both the 30% and 40% implant groups was similar to that of the sham surgery and placebo control group. Systemic administration of IRN is known to lower red blood cell counts [57]. This data is further evidence that the IRN is not entering the systemic circulation at significant concentrations to be toxic to red blood cells. A normal lymphocyte count is between 1000 and 4800 lymphocytes per microliter of blood. Figure 4E demonstrates that all groups have lymphocyte levels within the normal range. Systemic administration of IRN is known to decrease lymphocyte levels in the blood [58], which is further evidence that IRN blood levels after local administration are not high enough to cause systemic toxicity.

A normal neutrophil level is between 1500 and 8000 neutrophils per microliter. Systemic administration of IRN is known to lower neutrophil levels resulting in neutropenia [59, 60]. The neutrophil levels of both the Sham and Placebo control groups dropped from normal level on day 4 to below normal levels on day 14, with levels in the placebo group increasing to normal levels by day 45 and the sham surgery group reducing further. With the 30% group, the neutrophil levels were below normal on day 4, increasing to normal level by day 14 demonstrating that the IRN released from the 30% implants did not reach the systemic circulation of the mice in sufficient concentrations to cause neutropenia. However, with the 40% group, the neutrophil levels were below normal levels on day 4, increasing to normal levels by day 14, and then dramatically decreasing to below normal levels by day 45. The increased early release of IRN from both the 30% and 40% implants resulted in IRN reaching the systemic circulation of the mice in sufficient concentration to cause the neutrophil levels to drop below normal on day 4. With the 30% implants, the release of IRN after the initial early release was not sufficient to cause any further IRN to reach the systemic circulation so the neutrophil levels increased back to normal by day 14. However, the increased release from the 40% implants resulted in the IRN remaining in the systemic circulation causing the neutrophil levels to drop below normal by day 45.

The normal basophil range is between 0 and 300 per microliter of blood. The basophil levels for the sham, placebo, and 30% groups remained within the normal range, while for the 40% group, the basophil levels were at the high end of the normal range on day 4 and above the normal range at day 14, returning to normal range by day 45 (Fig. 4G). The basophilia in the 40% group at days 4 and 14 corresponds with the mild to moderate toxicity observed up to 6 days after implantation, returning to normal at day 14 and diminishing completely by day 16 (Fig. 3A). Both the Sham and Placebo groups had WBC levels within the normal range (Fig. 4H), while the WBC levels for the 30% and 40% groups fell below the normal range on day 4, returning to the normal range by day 14. Systemic administration of IRN is known to lower the WBCs; therefore, the increased early release resulted in IRN reaching the systemic circulation in sufficient concentrations to reduce the WBC. However, subsequent release was not high enough to result in IRN remaining in the systemic circulation at sufficient concentration to reduce WBC after 2 weeks. The normal eosinophil count should be less than 500 cells per microliter of blood. All groups had eosinophil levels within the normal range (Fig. 4I).

Both the BUN and creatinine levels were in the normal range (6 to 30 mg/mL and 0.7 to 1.2 mg/dL of blood, respectively) for all treatment groups (Fig. 5A and B). Systemic administration of IRN is known to reduce kidney function, with high creatinine blood levels associated with lower creatinine clearance levels in IRN patients [61]. Normal kidney function in the 30% and 40% w/w groups would suggest that the blood levels of IRN were below those needed to impair kidney function. The ALP and ALT levels of all groups were within the normal range of 44 to 147 and 4 to 36 U/L of blood, respectively (Fig. 5C). However, the AST levels in all groups were above the normal range (8 to 33 U/L of blood), with some of the highest levels detected in the sham surgery and placebo groups (Fig. 5C), which would suggest that it is not associated with IRN levels in the blood. Anesthesia and surgery have been shown to increase AST levels in the blood [62], and thus, the increased level in all groups is associated with the resection/implantation surgery.

Figure 6A shows the fold change of BLI values for the placebo, 30%, and 40% implant groups. All three groups had steady tumor growth from the day of tumor implantation (day − 6) to the day of tumor resection and implantation (day 0). After resection/implantation both the 30% and 40% groups demonstrated a continued reduction in tumor volume to below the baseline, whereas the placebo group had a significantly higher tumor volume that never went below the baseline and continued to increase until day 17. This is due to the early increased release of IRN from the 30% and 40% implants suppressing tumor growth. At day 4, the 40% group demonstrated a slow tumor regrowth, reaching the baseline (1.0-fold) by day 21, while the 30% group had a further decrease in tumor volume until day 10 followed by a slow increase in tumor volume, reaching the baseline at day 28. Fluc bioluminescence imaging of the mice (Fig. 6B) demonstrated that all groups had tumors 2 days before resection surgery. The placebo and 40% groups had tumor recurrence at day 4 and day 21 resulting in significant numbers of mice dying by day 38. The 30% group demonstrated no signs of tumor recurrence by day 38, with all mice still alive. The increase in tumor regrowth in the placebo and 40% groups resulted in a reduced survival with 100% of the mice dead by day 32 and 70, respectively, with the 30% group having 80% survival at day 148 (Fig. 6C). The BLI images of the whole brains of the dead mice in both the placebo and 40% groups demonstrate that they had tumor recurrence, while in the 30% group, none of the mice had tumor recurrence at the time of death/euthanization (Fig. 6D). The reduced efficacy of the 40% implants is due to their early onset of necrosis (Fig. 3B), as a result of their increased early release. This resulted in their gradual displacement within the resection cavity and the gap created filling with cerebral spinal fluid (CSF). Healthy tissue is stiffer than necrotic tissue [63, 64], which keeps the implants in direct contact with the cancerous tissue in the tumor margin. The more compliant necrotic tissue allows for the displacement of the implants, and thus, they are not in direct content with the cancerous tissue. With the 30% implants, there is direct delivery of the IRN into the cancerous tissue, whereas with the 40% implants, there is indirect delivery with the IRN first released into the CSF in the cavity, before reaching the cancerous tissue. This reduces the amount of drug delivered into the infiltrative cancerous tissue and facilitates faster clearance of the IRN from the tumor margin, as the drug in the CSF is washed away, resulting in faster tumor regrowth and death. The early onset of necrosis in the 40% implant group was between day 4 and 14 (Fig. 3B); thus, at this time point, the amount of IRN entering the cancerous tissue was reduced resulting in tumor recurrence beginning from day 10 (Fig. 6A, B, and D).

All implantable drug delivery devices need to be sterilized before they can be administered to patients. The use of gamma sterilization is the most suitable method for implantable drug delivery devices. However, gamma sterilization can have an impact on drug release and stability of PLGA-based devices via the formation of reactive radicals, which can impact the stability and release of the incorporated drug [51–54]. Therefore, it is important to evaluate the impact of gamma sterilization on the IRN content, distribution release, and crystallinity of the 30% implants. Furthermore, it is important that any pharmaceutical has an appropriate shelf-life. Therefore, we evaluated the impact of sterilization, real-time (20 °C and 60% relative humidity), and accelerated storage conditions (40 °C and 75% relative humidity) on the stability, release, distribution, and crystallinity of the IRN within the 30% implants. Figure 7A demonstrates that gamma sterilization has no significant (*P* = 0.65) impact on the IRN content. Furthermore, storage under both long-term and accelerated conditions for up to 12 months had no significant (*P* = 0.57) impact on the IRN content (Fig. 7A). Gamma sterilization had no significant (*P* = 0.21) impact on the accelerated dissolution rate of the IRN from the implants (Fig. 7C). Raman maps of their cross-section before and after gamma sterilization are presented in Fig. 7D. The Raman maps demonstrate that sterilization had no impact on the distribution of the IRN within the implants. Figure 7D and E demonstrate that neither sterilization nor storage under long-term and accelerated conditions had an impact on the crystallinity of the IRN within the implants.

The current standard of care for glioblastoma has not changed in 15 years and is extremely toxic for the patient while offering a dismal prognosis of 15-month survival. IRN has shown promise as a treatment for glioblastoma; however, its dose-limiting toxicities of neutropenia and diarrhea have limited its use to a second-line therapy. Here, we have demonstrated that the formulation of IRN into an implant for local delivery to the resection margin offers a promising treatment option for glioblastoma. The 30% and 40% implants showed only mild to moderate signs of local toxicity, with no signs of systemic toxicity such as neutropenia or diarrhea. Furthermore, the 30% implants had an 80% survival rate at day 148 with no sign of tumor recurrence in a PDX mouse resection model. Finally, gamma sterilization and storage under long-term and accelerated storage conditions had no impact on the IRN content, distribution, release crystallinity, or stability of the 30% implants. Other local drug delivery strategies for glioblastoma, such as gels [65], pastes [66], and nano- [67] and micro-particles [68], however, have significant limitation compared to the implant described here. For example, the gels and pastes are applied to the surface of the resection margin and can easily run and pool in the center of the cavity leaving areas of the margin untreated. Furthermore, they typically only allow for drug release over a few days and do not achieve the deep tissue penetration needed to reach the deep-seated tumor tissue associated with glioblastoma. Nano- and microparticles can be injected into the tumor tissue of the margin with the potential to provide sustained release to the deep-seated tumor tissue. However, given their small size and spherical shape, they tend to migrate away from the margin. The cylindrical shape of the implant described here allows for into to be implanted directly into the tumor tissue of the margin, providing sustained release, while remaining in place at the margin. Thus, the potential of the 30% w/w IRN-loaded implant described here as a safe and effective treatment for glioblastoma highlights the urgency of its evaluation in clinical trials.

## Data Availability

The datasets generated during and/or analyzed during the current study are available from the corresponding author on reasonable request.
